# Applying Self-Supervised Representation Learning for Emotion Recognition Using Physiological Signals

**DOI:** 10.3390/s22239102

**Published:** 2022-11-23

**Authors:** Kevin G. Montero Quispe, Daniel M. S. Utyiama, Eulanda M. dos Santos, Horácio A. B. F. Oliveira, Eduardo J. P. Souto

**Affiliations:** Computer Institute, Federal University of Amazonas, Manaus 69080-900, Brazil

**Keywords:** self-supervised learning, representation learning, emotion recognition, physiological signals, wearable sensors

## Abstract

The use of machine learning (ML) techniques in affective computing applications focuses on improving the user experience in emotion recognition. The collection of input data (e.g., physiological signals), together with expert annotations are part of the established standard supervised learning methodology used to train human emotion recognition models. However, these models generally require large amounts of labeled data, which is expensive and impractical in the healthcare context, in which data annotation requires even more expert knowledge. To address this problem, this paper explores the use of the self-supervised learning (SSL) paradigm in the development of emotion recognition methods. This approach makes it possible to learn representations directly from unlabeled signals and subsequently use them to classify affective states. This paper presents the key concepts of emotions and how SSL methods can be applied to recognize affective states. We experimentally analyze and compare self-supervised and fully supervised training of a convolutional neural network designed to recognize emotions. The experimental results using three emotion datasets demonstrate that self-supervised representations can learn widely useful features that improve data efficiency, are widely transferable, are competitive when compared to their fully supervised counterparts, and do not require the data to be labeled for learning.

## 1. Introduction

Emotion recognition remains one of the most researched topics in the area of affective computing [1]. In this research area, many systems have been developed to model and interpret the affective states of humans [2,3,4]. As emotion is considered a physiological and psychological expression associated with individuals’ moods and personalities, these systems use sensing technologies, usually micro-sensors integrated into wearable devices, and computational models generated from machine learning techniques are used to analyze the physiological signals and infer or quantify human emotions [5].

A wide variety of physiological signals can be collected non-invasively from wearable devices, such as electrocardiography (ECG), electroencephalography (EEG), galvanic skin response (GSR), temperature (TMP), and electromyography (EMG) signals. Due to the large amount of data that can be obtained by these devices, the complexity to infer affective states from these signals still represents a challenge. Different machine learning (ML) models based on different algorithms, such as k-nearest neighbors (k-NN) [6], support vector machines [7], and deep neural networks [8], have been evaluated for this task.

In most existing solutions, supervised machine learning has been the conventional training paradigm for the models proposed for emotion recognition systems [9]. Despite high classification performance rates, these models require a large amount of annotated data, so the cost of annotating the data becomes a major bottleneck for the development of pattern recognition systems, especially in healthcare, where data annotation requires even more specialized knowledge (e.g., physicians), and recent privacy concerns hinder the use of real user data.

These needs have motivated research in which learning about data representations is performed in a self-supervised manner [10]. The area of self-supervised learning (SSL) describes a class of methods that allow networks to take advantage of unlabeled training data and learn to extract meaningful representations without any manual annotation [11]. The self-supervised learning approach has shown promising results when applied to images [12] and text [13]. In this learning approach, a part of the neural network input is used as a supervising element; the result of which is a model or representation that can be used for solving the original modeling problem.

The representations obtained in this way have demonstrated more effective, generalizable, and transferable results for final tasks for which labeled data are limited or costly to obtain. This makes the self-supervised learning task a promising approach for solving problems in healthcare, in which the volume of unlabeled data generated by numerous medical devices and services is immeasurable. Despite this, SSL has been little explored in healthcare, especially for emotion recognition.

In this paper, an overview of the main concepts in the field of emotion recognition, including how emotions are represented and measured, is provided. A description of the main physiological signals and techniques that are often applied to infer affective states is also supplied. Furthermore, a discussion on how the development of systems in application domains with few labeled data moves towards the use of self-supervised learning is presented. In order to expand the reader’s understanding of the employment of self-supervised learning, an example of an application of self-supervised representation learning for emotion recognition is given. Self-supervised and fully supervised training of a convolutional neural network designed to recognize emotions were experimentally analyzed and compared, and the effectiveness of pre-training the network with self-supervision to improve model capability was investigated. The experimental results using three emotion datasets (AMIGOS [14], DREAMER [15], and SWELL [16]) demonstrated that self-supervised representations learn widely useful features that improve data efficiency, are widely transferable, and are competitive compared to their fully supervised counterparts, as well as not requiring data to be labeled for learning.

In summary, our main contributions are as follows:

- A comprehensive review of recent research on supervised, semi-supervised, and self-supervised learning in human emotion recognition using physiological signals is presented.
- An ECG-based emotion recognition use case that implemented multi-task self-supervised learning is proposed. A convolutional neural network is trained to learn generalizable features without labeled data using the signal transformation recognition problem as pretext tasks.
- In three publicly available datasets, the results show that our self-supervised model is comparable to or better than an emotion recognition model learned through fully supervised training (i.e., from scratch) for the same network architecture.

The remainder of the article is organized as follows. Section 2 presents a contextualization of the emotion recognition area and the main physiological signals used. Section 3 describes the main machine learning approaches applied to the emotion recognition problem. A review of recent works applying deep learning in the area of affective computing is presented in Section 4, including works applying self-supervised learning. In Section 5, the methodology of self-supervised learning is explained in more detail, and an example application of this approach for emotion recognition is presented. In addition, the supervised and self-supervised approaches are compared, and the achieved performances are analyzed. Finally, in Section 6 and Section 7, the advantages and limitations of the self-supervised approach are discussed, and possible future work is presented.

## 2. Emotion Recognition

Emotions are affective states that influence behavior and cognitive processes. They appear as a result of external or internal stimuli and are accompanied by physical and physiological reactions. In the following sections, the definition of emotions, models for representing emotions, as well as the main characteristics of the physiological signals used to measure affective states are presented and briefly described.

### 2.1. Defining Emotions

Human emotions are complex and multifaceted phenomena. This is due to the numerous proposed theories and perspectives by which emotions are studied [17]. In general, emotions have been described as a response to events or stimuli, have a short duration, and correspond to a coordinated set of responses, which include verbal, behavioral, physiological, and neural responses [18,19].

Three distinct components can be observed in an emotional response: subjective experiences, physiological responses, and behavioral responses. Emotions start from a stimulus that produces a subjective experience, in which a wide variety of elements (e.g., culture, education, previous experiences, and personality) can determine a person’s perception and responses. Subjective experiences can vary in intensity from person to person, as well as provoking many emotions in a single individual. Based on subjective experience, behavioral responses are the expressions of emotion, such as a smile, a laugh, a scream, and other reactions. For example, fright in response to an unexpected and intense stimulus is a universal reflex that involves multiple motor actions, which include tension in the neck and back muscles and blinking of the eyes. The physiological responses, however, are the results of autonomic nervous system reactions to emotional experiences. The autonomic nervous system controls the body’s involuntary responses, such as breathing, heartbeat, and pupil movement, among others. For example, in response to a stressful stimulus, substances such as adrenaline and cortisol are rapidly released in the body and prepare the individual for a “fight or flight” reaction.

### 2.2. Emotion Representation Models

**Figure 1:**
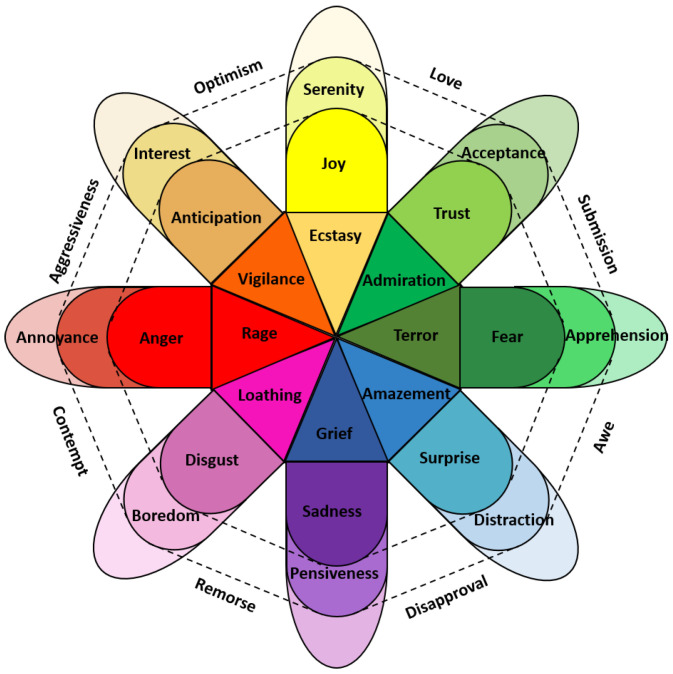
Plutchik’s color wheel showing the eight primary emotions.

From a layman’s point of view, it is easy to determine whether someone is experiencing or expressing a specific emotion (e.g., happiness, fear). However, determining or measuring a person’s emotional state is one of the most debated problems of affective science [20]. This is because of the different perspectives of representing emotions. There are two common perspectives for representing an emotion: dimensional and discrete. In the discrete perspective, each emotion corresponds to a unique and universal profile in experience, physiology, and behavior. Ekman [21] argued that all people in the world can express and recognize their emotions using six basic emotions: sadness, happiness, surprise, fear, anger, and disgust. Although many psychologists have accepted the theory of basic emotions, there is no consensus on the precise number of basic emotions. Robert Plutchik [22], for example, proposed eight primary emotions: anger, fear, sadness, disgust, surprise, anticipation, confidence, and joy, and arranged them in a colored wheel, as shown in Figure 1. Other research argues that other emotions can be considered from the intensity or combination of the basic emotions. Zenonos et al. [23] presented an approach to distinguish eight different emotions and moods (excited, happy, calm, tired, bored, sad, stressed, and angry). In the view of some researchers, discrete models are unable to capture some human emotions [24]. Despite this, discrete emotion models are widely used because of their simplicity and high degree of interpretability.

**Figure 2:**
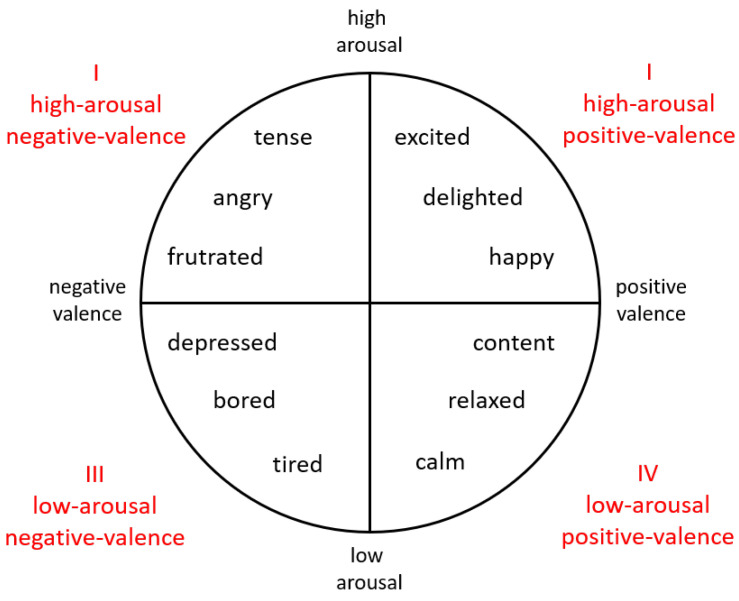
Russell’s two-dimensional circumplex model showing the distribution of emotions.

In the dimensional perspective, there are some fundamental dimensions that organize emotional responses [25]. Dimensional studies of emotions originated from W. M. Wundt [26], who proposed that emotions can be defined using three independent dimensions: pleasure–displeasure, excitement–inhibition, and tension–relaxation axes. J. A. Russell [25] introduced a circumplex model, in which emotions can be distributed in a circular dimensional space that is composed of two independent dimensions: arousal and valence. The valence dimension indicates the perception of how positive or negative the current affective state is. In the arousal dimension, the state is classified in terms of the level of activation, i.e., it measures the intensity of the emotion. As shown in Figure 2, arousal and valence represent the vertical and horizontal axes, while the center of the circle equals a medium level of arousal and neutral valence. In this model, emotional expressions can be illustrated at any level of arousal and valence or defined from four regions (quadrants).

### 2.3. Measuring Emotions

**Figure 3:**
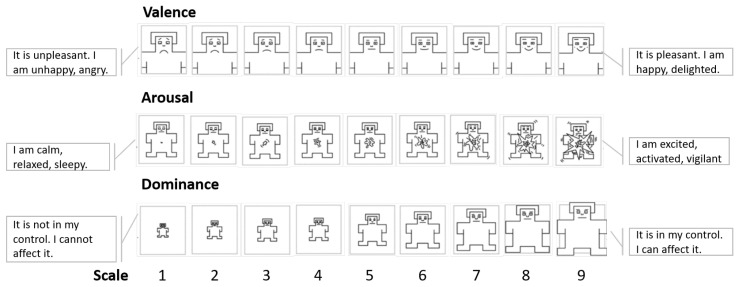
Self-assessment manikin (SAM) questionnaire and its scales, respectively, valence, arousal, and dominance.

Measuring emotions can be accomplished from the three components observed in an emotional response. Subjective experience, for example, can be captured through self-assessment questionnaires (self-reports). Specifically, self-reports of recent emotional experiences are more valid than self-reports of experiences distant in time [27]. Figure 3 shows an example of a self-assessment manikin (SAM) questionnaire designed to capture emotional experiences from a dimensional perspective (arousal, valence, and dominance).

Observing the behavior, emotions can be gleaned from vocal characteristics, facial expressions, and body gestures. Human speech is one of the main forms of human expression [28]. In addition to conveying the desired information from the sound of words, the speaker also shares information via tone of voice, energy, speed, and other acoustic properties, which help the receiver gauge the intentions and emotions of that communication. On the other hand, facial expressions and body gestures are the most common ways of identifying [29] emotion. Research from the literature supports the existence of a universally recognized set of facial expressions for emotions such as happiness, surprise, fear, sadness, anger, and disgust [29]. In addition, research based on body movement, posture, and gestures have grown in recent years, given the possibility of recognizing emotions at a distance.

Contrastingly, by observing the physiological responses of an emotional episode, emotions can be obtained from physiological signals or indications of autonomic nervous system activation [30]. Electroencephalography (EEG or EKG), electrocardiography (ECG), electrodermal activity (EDA), galvanic skin response (GSR), and electromyography (EMG) signals are the most common physiological signals that can be used to measure emotions. The next section will discuss in more detail the inference of emotional states by means of these signals.

### 2.4. Relationship between Physiological Signals and Emotions

Scientific studies related to the field of psychology point out that human emotions and physiological responses are clearly interconnected [31,32]. For example, some negative emotional states, such as fear and anxiety, can lead an individual to exhibit strong physiological indicators such as sweating, a dry mouth, or feeling unwell [8,33,34,35]. Another example is the state of happiness, in which the response pattern is characterized by increased cardiac activity, vasodilation, increased electrodermal activity, and increased respiratory activity [30].

The expression of emotions through physiological responses is a natural process, usually unconscious and controlled by the central nervous system, which makes it difficult for the subject to fake or mask his/her emotional reactions. Thus, the inference of emotions through physiological signals has advantages compared to inference from subjective experiences or behavioral responses [36,37].

Among the existing physiological signals, the main physiological signals and techniques often applied to infer affective states are presented in detail below.

#### 2.4.1. Electroencephalography

Electroencephalography (EEG) measures the electrical activity of the brain and is indicated for identifying neurological changes [38]. Several features of the EEG signal, such as the alpha and beta bands, are useful for identifying positive self-evaluative emotions such as gratitude, inspiration, and pride; the theta and gamma bands are used to characterize pleasure emotions such as amusement, interest, and joy [39]. For this reason, EEG signals are used by many studies to detect an individual’s emotional responses to stimuli [40]. Krishna et al. [41], for example, proposed the use of EEG signals to identify the expressions of emotion by physically disabled or immobilized people. Zhang et al. [42] proposed a method for selecting the best channels of the EEG signal to identify the emotions of joy, fear, sadness, and relaxation. Other studies seek to evaluate different emotions and discuss which types of stimuli (visual, audio, or audiovisual) are best for establishing emotions from EEG signals [43,44].

#### 2.4.2. Electrocardiography

Electrocardiography (ECG) is a record of the electrical activity generated by the heart during a time interval [45]. In the health field, it is an effective and non-invasive tool, which, in addition to providing data to diagnose abnormalities present in the heart, can also be used to identify the emotional states of individuals [46], since emotions can produce variations in the signals of the ECG [37].

The main parameters of the electrocardiography signal, such as the P, Q, and T waves, QRS complex, and QT/QTc, are often used in the analysis of an individual’s cardiac activity. Most of the studies related to ECG-based emotion recognition focus on the evaluation of the duration and amplitude of the QRS complex [1]. For example, C. Jing [47] analyzed features extracted from the QRS complex and showed that sadness can be recognized more easily and accurately than the emotion of joy. Uyarel et al. [48] analyzed the dispersion of the QT/QTc parameter and proved that this physiological measurement can be used as a marker to recognize intense anger.

One disadvantage of using the ECG signal is that it is very sensitive to noise and is usually obtained in clinical spaces when the patient is in a calm state.

#### 2.4.3. Electrodermal Activity

Electrodermal activity (EDA) is the change in electrical properties of the skin with respect to sweat excretion, obtained by the continuously varying electrical characteristics of human skin [1]. By applying a small electric current, the variation of skin conductance (SC) can be measured non-invasively. In addition, the galvanic skin response (GSR) is the measurement of the variation in SC in response to sweat excretion activity. The GSR is often referred to as EDA or SC [49]. This is a measurement that cannot be controlled voluntarily and is established as an important variable for measuring emotional arousal [50].

Emotional changes induce sweat reactions, which are mainly noticeable on the surface of the fingers and soles of the feet. The sweat reaction causes a variation in the amount of salt in human skin, and this leads to a change in the electrical resistance of the skin [51]. The conductance of the skin is mainly related to the level of excitation: if the level of excitation increases, the conductance of the skin also increases. For this reason, some research seeks to use the EDA signal to identify diseases and changes in affective states such as stress, excitement, frustration, anger, and pain [52,53,54,55]. Compared to EEG and ECG, GSR requires a smaller quantity of electrodes for measurement, which facilitates the use of wearable devices and the definition of emotional states when a person engages in normal activities [1]. However, like the other techniques, its accuracy is also affected by motion artifacts.

#### 2.4.4. Electromyography

Electromyography (EMG) is employed to measure muscle electrical activity for the stimulation of a nerve or muscle [56]. EMG is used in many areas of science, including in the assessment of neuromuscular health [57], assessment of muscle activation for sports [58], gait analysis [59], assessment of muscle fatigue [60], in the actuation and control of prostheses and exoskeletons [61], and in the field of psychology [62].

In the field of emotion recognition, EMG is used to find the relationship between cognitive emotions and physiological reactions [63]. Most of the works using EMG for the recognition of emotional reactions focus on the analysis of facial expressions. For example, Kim et al. [64] explored the use of facial EMG and EEG signals for the classification of the emotions of happiness, surprise, fear, anger, sadness, and disgust. Mithbavkar et al. [65] developed a dataset for emotion recognition based on data collected through electromyograms using dance to stimulate emotional responses such as astonishment, awe, humor, and tranquility. While Wioleta [66] proposed feature extraction from EMG, blood pressure, and GSR measurements for the detection of the emotional stages of happiness, sadness, anger, hatred, and respect.

Just as in procedures that require contact measurement, such as EEG and ECG, EMG affects people’s comfort levels and creates limitations for its continuous use. However, it is a very good technique for detecting strong emotions, since drastic changes in valence and intensity of arousal produce changes in facial expressions [67].

#### 2.4.5. Heart Rate Variability

Heart rate variability (HRV) represents the variation in the time interval between consecutive heartbeats [68]. Heart rate variability is regulated by the autonomic nervous system, specifically by sympathetic nerves, which speed up the heart rate, and parasympathetic nerves, which slow down the heart rate. Changes in heart rate are influenced by emotions, stress, and exercise [68,69]. HRV measurements are used to monitor affective states such as anxiety, anger, fear, stress, and relaxation [70] or aid in the detection or treatment of psychiatric illnesses such as depression [71], anxiety [72], and drug addiction [73]. Thanapattheerakul et al. [74], for example, showed that feeling sad when induced by crying tends to increase HRV. This feature of HRV shows that the intensity and context in which stimuli are presented can affect the detection of emotional stages.

HRV measurements are commonly obtained from the ECG signal, which provides information on the variation of the RR interval in relation to time. However, they suffer from the sensitivity and noise problems already mentioned regarding the use of ECG. One alternative that has been widely used, mainly by the immense proliferation of smartwatches, is photoplethysmography (PPG). This technique is used to detect changes in blood volume in microvascular tissues; its operation is via a photodetector and a light source, which illuminates the tissue, and the photodetector measures the small variations in the reflected light [68]. There are a variety of studies that prove the advantages of using this technique for HRV signal extraction when compared to ECG [75,76]. Besides the usual PPG approach already mentioned, there is also the remote one, by which it is possible to retrieve the cardiovascular pulse waveform by measuring the variations in the light emitted remotely in the environment by means of computer vision systems [68]. This approach increases the comfort level of the person during the measurement procedure, but increases the noise in the signal, thus requiring advanced signal processing and analysis systems.

Emotion recognition systems, such as those presented in this paper, can be used to infer the emotional states of humans. Nonetheless, the analysis of high-dimensional patterns and correlations of the above physiological signals would be practically impossible without computers and computational methods such as machine learning [77].

## 3. Towards Self-Learning Systems for Emotion Recognition

Inferring a person’s emotional state using physiological data collected from wearable devices is challenging. Typically, machine learning models are built from features extracted from the raw data of the collected signals, which are usually determined based on knowledge of the problem domain. For example, extracted statistical measures, such as the kurtosis and asymmetry of the ECG signal, are used to detect stress [45].

This procedure of designing sophisticated feature extraction techniques or creating them manually is called feature engineering and depends on an expert. Handcrafted approaches of feature extraction are usually unable to extract high-level discriminative information from raw data due to different problems such as learning variety from complex data, overcoming the noises present in the signals, and dealing with high intra-class diversity [78].

Deep learning provides a set of methods to overcome these limitations and is one of the most successful approaches to learning high-level representation from complex raw data, and it has recently made remarkable progress, especially in emotion recognition applications [34]. In general, the methods used are based on supervised learning, in which different architectures are trained, usually based on convolutional neural networks (CNNs) and recurrent neural networks (RNNs) [5]. Nevertheless, the design of classification methods based on this approach requires a large amount of data and annotations (labels) for training the networks.

In a typical supervised learning setting, deep neural networks are dependent on the training database (samples and labels), which means that performance and generalization are typically limited by the size of the database. However, acquiring such a training database can be expensive and time-consuming, especially in healthcare since labeling samples requires even more specialized knowledge, and recent privacy concerns make it difficult to use real user data.

Given these problems, current research focuses on developing methods that do not require or only require few labeled data. This has led to advances in the field of machine learning such as the introduction of transfer learning methods, semi-supervised learning, and self-supervised learning [10].

Transfer learning is a popular approach for circumventing the limitation of labeled datasets. Transfer learning attempts to improve traditional machine learning by transferring the knowledge learned on one or more source tasks and using it to improve learning on a related target task. To do this, the model is trained for a similar problem for which a labeled database exists; hence, the knowledge gained serves as prior training for the target model. In this way, transfer learning can help to reduce costs and, at the same time, improve performance. Despite this benefit, this type of learning only works well if the original and target tasks are related [79].

Another alternative adopted by designers of pattern recognition systems to overcome data scarcity is to train the algorithm based on a combination of labeled and unlabeled data; this approach is known as semi-supervised learning. Typically, this combination will contain a very small amount of labeled data and a very large amount of unlabeled data. The basic procedure involved is that, first, the developer clusters similar data using an unsupervised learning algorithm and will then use the existing labeled data to label the rest of the unlabeled data. In general, the unlabeled samples are assumed to belong to the same or similar distributions as the labeled samples.

The area of self-supervised learning (SSL) describes a class of methods that allow networks to take advantage of unlabeled training data and learn to extract meaningful representations without any kind of manual annotation [11]. In this learning approach, substitute tasks (also known as pretexts) are defined for which supervision can be acquired from the data themselves. This makes the self-supervised learning task a promising approach for solving problems in healthcare, since the volume of unlabeled data generated by the numerous medical devices and services is enormous.

Although SSL is a promising approach for learning representations from a huge amount of unlabeled data, it has been little explored in the area of emotion recognition. In the following section, a review of some recent works that propose deep neural network architectures to recognize emotions using physiological data is presented.

## 4. Related Works

In many healthcare applications, data collection is becoming increasingly less expensive, mainly due to the employment of wearable devices; however, data annotation still involves manual and skilled labor and is therefore expensive. In this section, recent work to recognize emotions that uses large amounts of labeled physiological data (supervised approach) is presented, as well as work that attempts to reduce the cost of learning new models using only a small proportion of labeled data (semi-supervised approach) or employs a strategy by which the supervised task is created from the unlabeled data (self-supervised approach).

### 4.1. Supervised Deep Learning

Supervised learning consists of learning models built from training samples for which each sample has a label. This label is usually defined by an expert and is used by the model to learn to make correct decisions. Although the process of data annotation and labeling is costly, most emotion recognition work using physiological data adopts this approach.

Radhika and Oruganti [80] investigated the influence of multimodal data fusion on convolutional neural network-based (CNN) models for subject-independent stress detection via the physiological signals of electrocardiograms (ECGs) and electrodermal activities (EDAs). The authors extracted features in the time and frequency domain from ECG and EDA signals made available by the ASCERTAIN and CLAS datasets. Different stress detection models were generated from the combination of the 50 most-relevant features. The authors performed three sets of experiments on each database. On the ASCERTAIN database, using a model built only with the characteristics extracted from the ECG signal, they obtained 71% accuracy; with the model generated from the characteristics of the EDA signal, they obtained 68.7% accuracy; using the characteristics extracted from the ECG and EDA signals, they obtained an accuracy rate of 75.5%. On the CLAS database, the accuracy rates were 71.8%, 64.4%, and 69.9% for the models generated with the ECG, EDA, and ECG+EDA signals, respectively.

Hsu et al. [81] presented a method for human emotion recognition based on ECG signals. The authors proposed a music induction method to induce the participants’ real emotional states and collect the ECG signals. The physiological features of the ECG were extracted from the time and frequency domain. Then, the proposed method uses a sequential forward floating selection-kernel-based class separability-based feature selection algorithm and generalized discriminant analysis to select the most relevant features associated with emotions and reduce the feature space, respectively. Positive/negative valence, high/low arousal, and four types of emotions (joy, tension, sadness, and tranquility) are recognized using least-squares support vector machine (LS-SVM) recognizers. Experimental results with data from 31 participants showed that the proposed method obtained classification rates of 82.78% for valence, 72.91% for arousal, and 61.52% for the four discrete emotions.

Montesinos et al. [82] proposed a multimodal machine learning method to recognize acute stress based on biomarkers extracted from physiological signals, which were acquired from the Shimmer3 ECG Unit wearable devices and the Empatica E4 wristband. Features extracted from the physiological signals ECG, blood volume pulse (BVP), skin temperature (SKT), respiration (RSP), and EDA were used to generate stress detection models using the k-NN, decision tree, and random forest classifiers. Experimental results with 30 participants, induced to stress and non-stress states, showed that it was possible to detect acute stress episodes with an accuracy of 84.13% for an unseen test set using the proposed multimodal machine learning and sensor data fusion techniques.

Bobade and Vani [83] proposed stress recognition through physiological signals using shallow and deep machine learning algorithms. Data from different sensors such as acceleration, electrocardiogram, pulse blood volume, body temperature, respiration, electromyogram, and electrothermal activity data were used to classify three physiological states: fun, neutral, and stress state. Evaluations of the methods treating the problem as a binary problem (stress and non-stress) were also carried out. During the study, using machine learning techniques, accuracies of up to 81.65% and 93.20% were achieved for binary and three-class classification problems, respectively, and using deep learning, they achieved accuracy up to 84.32% and 95.21%, respectively.

Yang et al. [84] proposed a platform to recognize emotions based on two input systems: an emotion recognition system based on electroencephalogram (EEG) signals and a system based on ECG and PPG. The first system has as the input the spectrogram features obtained from the short-time Fourier transform of the EEG, while the second uses a multimodal implementation based on the statistical and intrinsic features of the ECG and PPG signals for the classification of three emotion states: happiness, anger, and sadness. The first model was evaluated with the leave-one-subject-out technique and obtained 76.94% accuracy, and the second with the subject-dependent technique showed 76.80% accuracy.

Behinaein et al. [85] proposed a novel architecture for stress recognition via ECG that consists of a deep neural network with convolutional layers and a transformer mechanism. In more detail, the architecture is made up of three subnets: a convolutional subnet, a transform encoder, and a fully connected (FC) subnet. Experiments on two databases using leave-one-subject-out validation demonstrated that, by fine-tuning the model with only a fraction of the test data (10%), it achieved optimal results, an accuracy of up to 71.4%, which is comparable to or better than state-of-the-art models for ECG-based stress detection.

Furthermore, Siddharth et al. [86] proposed a hybrid deep neural network for emotion recognition from ECG and PPG signals. For this, features from these signals were extracted and fused with deep-learning-based spectrogram features. Experiments showed that the hybrid method can set up benchmarks for the AMIGOS and DREAMER datasets.

### 4.2. Semi-Supervised Learning

While labeling data is expensive, collecting physiological data to recognize emotions is relatively easy and part of the clinical routine. The use of wearable health devices (e.g., fitness trackers, ECG monitors, blood pressure monitors, and biosensors) has further facilitated this collection process. Therefore, using these unlabeled data can not only improve the performance of classifiers, but also decreases the cost of designing emotion recognition systems. In this context, recent works have proposed semi-supervised methods for emotion classification based on the combination of supervised and unsupervised approaches.

Zhang et al. [87] proposed a semi-supervised approach for recognizing emotions using a deep recurrent autoencoder (AE). The method was trained in an unsupervised manner, and its encoder component was trained simultaneously in a supervised manner. The authors evaluated the proposed method using the SEED database and compared the obtained results with other works in the literature. The evaluation showed that the proposed method consistently achieved better results than other methods when few labeled samples were used (3%, 5%, and 10%).

Peng et al. [88] proposed a self-weighted, semi-supervised classification (SWSC) model that is capable of recognizing emotions from EEG signals. The SWSC incorporates a self-weighted variable that assigns weights to features according to their relevance in different emotion recognition sessions using combinations of labeled and unlabeled data. Such an approach allows the proposed model to identify the frequency bands and EEG channels, which are considered stable for affective pattern recognition. Experimental results demonstrated that the self-weighting approach can effectively improve emotion recognition performance, and it achieved an average accuracy of up to 81.52%.

Luo et al. [89] presented a model to recognize affective states (valence, arousal, and dominance) based on a stacked denoising autoencoder (SDA) architecture with unsupervised pre-training followed by supervised fine-tuning. This semi-supervised learning architecture is used to extract emotional data representations from physiological signals without any human intervention. Experiments were conducted using manually extracted features (handcrafted features) and with data augmentation. The results showed that the proposed SDA overlaps with the other three deep network models evaluated.

### 4.3. Self-Supervised Learning

Self-supervised methods have been successfully employed in computer vision applications and have been the natural choice to deal with the scarcity of labeled data [10]. However, the use of transformations of physiological signals collected from different sensors to automatically generate labels has been little explored in the area of emotion recognition. Sarkar and Etemad [5] presented a self-supervised multitasking approach for emotion recognition based on ECG signals. The proposed solution consists of two steps: self-supervised training and an emotion recognition network. First, the network learns the abstract high-level representations from the unlabeled ECG data. For this, the authors used six different signal transformations for the collected ECG signals. Then, the six transformed signals together with the original signals are used to train a convolutional neural network to recognize the transformations. In the next step, the weights of the self-learning network are transferred to an emotion recognition network, so that the convolutional layers are kept frozen, and the dense layers are trained with labeled ECG data. Experimental results using four datasets (AMIGOS, WESAD, DREAMER, and SWELL) showed that the proposed model had higher accuracy rates compared to the same network when trained in a fully supervised manner.

Zhang, Zhong, and Liu [90] proposed a framework for self-supervised data augmentation in order to recognize emotions from EEG signals. The framework, named GANSER, is composed of a network based on an adversarial augmentation network (AAN) and a multi-factor training network. The AAN employs a masking transformation operation to mask parts of the EEG signals and force a generative adversarial network to generate EEG signal samples based on the remaining parts. Then, the simulated EEG signals are used in training emotion recognition models. The experimental results using three datasets showed that the proposed framework solves the data sparsity problem and outperforms the evaluated existing methods.

Rodriguez et al. [91] proposed a transform-based model to process ECG signals, in which this mechanism is used to build contextualized representations of the signal, which give more importance to the relevant parts to predict emotions. The authors employed self-supervised learning to solve the problem with a small amount of labeled data. This approach allowed several unlabeled datasets of the ECG signal to be used to pre-train the emotion model, then the model was optimized for emotion recognition on the AMIGOS database. The experiments indicated that the proposed model achieved better results when compared to the works in the literature with the supervised approach using the same database. The best result was obtained by pre-training the model to predict two classes: 88% accuracy for arousal prediction and approximately 83% for valence prediction.

## 5. Understanding Self-Supervised Learning with an Example

Self-supervised learning has become one of the main options for creating scalable models in various application domains, including healthcare. The main advantage of the self-supervised learning approach lies in the ability of a system to learn without manual annotation. In this section, an example of an application of self-supervised representation learning for emotion recognition is described, with the goal of demonstrating the advantages of developing future methods that apply this new approach.

### 5.1. Overview

**Figure 4:**
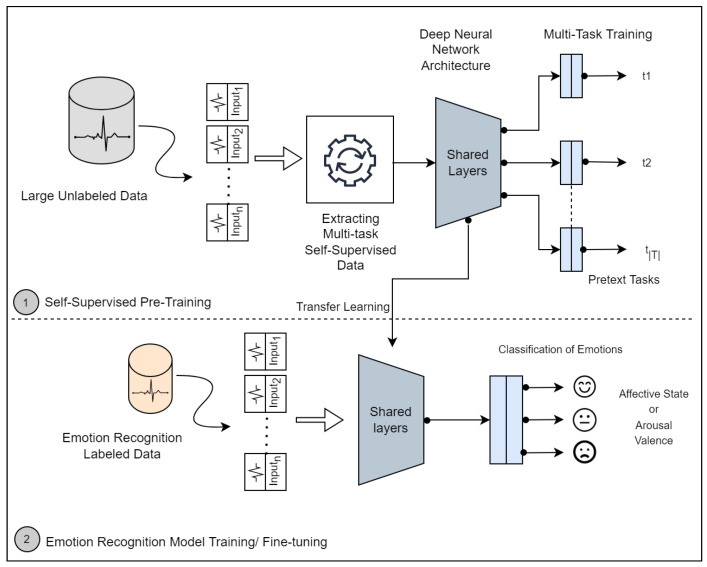
Overview of the self-supervised approach for emotion classification. The idea is to train a deep neural network to recognize signal transformations (i.e., pretext tasks), as shown in Step 1. The learned knowledge is transferred to an emotion recognition model (Step 2) to improve the detection rate.

Self-supervised learning is an innovation of unsupervised learning, which has recently been studied with the goal of learning high-level representations from unlabeled data and alleviating the dependency of large labeled data. In this learning approach, the goal is to learn a general-purpose representation based on a self-supervised deep network and use that representation later to solve the target task. The process consists of two steps, as illustrated in Figure 4.

The first step (1) consists of training the self-supervised deep network $M_{\theta }\left(.\right)$ designed to solve multiple pretext tasks. Therefore, a set of distinct transformations is defined as ${\left\{J_{t}\left(.\right)\right\}}_{t\in T}$, where $J_{t}\left(.\right)$ is a function that applies a particular signal transformation technique *t* to time series (signal) $x\in R^{2}$ to yield a transformed version of the signal $J_{t}\left(x\right)$.

The network $M_{\theta }\left(.\right)$ has a common trunk (shared layers) and individual head for each pretext task; it takes an input sequence and produces a probability of the signal being a transformed version of the original, i.e., $P\left(J_{t}|x\right)=M_{\theta }\left(x\right)$. Therefore, given a set of unlabeled signals, we can automatically construct a self-supervised labeled dataset $D={\left\{\left\{J_{t}\left(x_{i}\right),True\right),\left(x_{i},False\right)\right\}}_{t\in T}{\}}_{i=1}^{m}$.

Hence, given this set of *m* training instances, the multi-task self-supervised training objective that a model must learn to solve is:

(1) $$\min_{\theta }\sum_{t\in T}\psi_{t}\left[-\frac{1}{m_{t}}\sum_{i=1}^{m_{t}}\left(y_{i}^{t}\log\left(M_{\theta }\left(x_{i}^{t}\right)\right)+\left(1-y_{i}^{t}\right)\log\left(1-M_{\theta }\left(x_{i}^{t}\right)\right)\right)\right],$$

where $y_{i}^{t}$ is the automatically generated label, $M_{\theta }\left(x^{t}\right)$ is the predicted probability of *x* being a transformed version *t*, θ are the network’s learnable parameters, $m^{t}$ represents the number of instances for a task, and $\psi_{t}$ is the loss-weight of task *t*.

With the model pre-trained in a self-supervised way, the second step (2) consists of reusing the self-supervised representation to specialize a model for a target task. In the following example, a deep neural network $N_{\theta }$ is designed to classify emotions. This network has a common trunk architecture that was used in self-supervised learning and shares the same learned parameters. The common trunk (shared layers) is frozen, as with most transfer learning methods, and only the head is trained from scratch.

The model is trained with the true emotion labels $y_{i}$ for emotion classification; it takes an input sequence $x_{i}$ and produces a probability vector of emotion classes. Finally, the training objective is minimizing the cross-entropy loss:

(2) $$\min_{\theta }\sum_{i=1}^{C}y_{i}\log N_{\theta }\left(x_{i}\right),$$

where *C* is the total number of emotion classes.

### 5.2. Self-Supervised Task: Signal Transformations

Predicting the rotation of an image [92] or predicting a word by considering the surrounding words [93] comprises some commonly used pretext tasks in the computer vision and natural language processing fields, respectively. An example of a pretext task for physiological signal applications is to differentiate the original signal from its perturbed or transformed version. In this example, six signal transformations are used for self-supervising a network. These signal transformations have already been used in human activity recognition problems [94] and for emotions [5]. The transformations used in this work are summarized in Table 1 below.

The main motivation for using the pretext tasks defined above is to allow the network to capture the main characteristics of the signal. More specifically, for the network to successfully recognize whether the signal is transformed or not, it must learn possible distortions that the signal may suffer. In practice, this knowledge will be useful in the final task of the self-supervised approach for detecting emotions.

### 5.3. Network Architecture and Implementation

**Figure 5:**
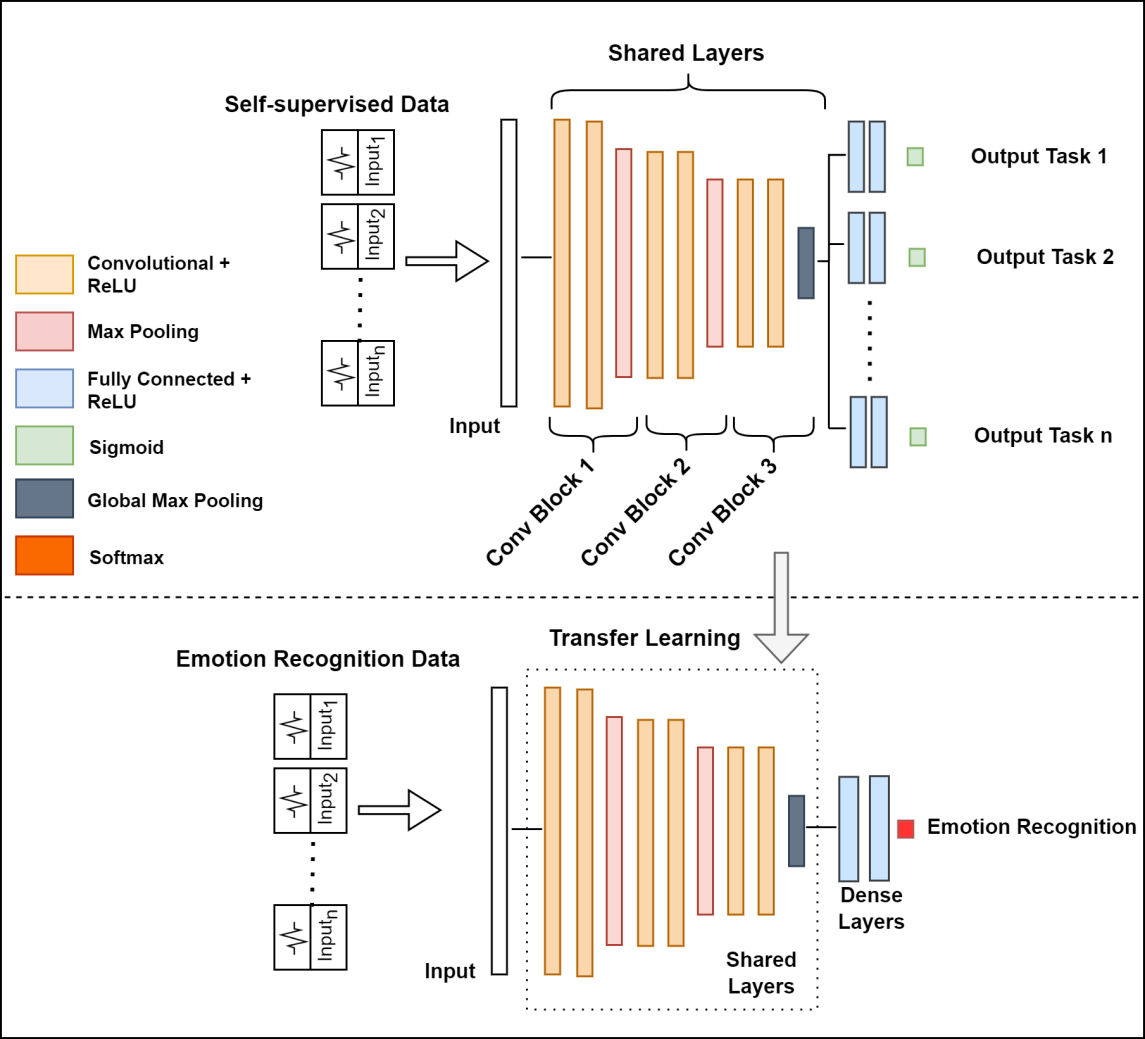
The architecture of the implemented convolutional neural network. The first stage is pre-training with 7 simultaneous tasks. The second stage is supervised training from the representation obtained in the self-supervised pre-training.

A convolutional neural network model was implemented to learn to classify signal transformations in the self-supervised pre-training phase. Figure 5 illustrates the common trunk containing three blocks that contain two 1D convolution layers with feature mappings of 32, 64, and 128, kernel sizes of 32, 16, and 8, respectively, and 1 stride. An L2 kernel regularizer with a rate of 0.0001 was used in the convolution blocks and fully connected dense layers. Global max pooling was used after the last convolutional layer to aggregate all high-level discriminative features. In addition, each specific path was composed of two fully connected dense neural layers with 128 hidden units, followed by an output layer with the sigmoid activation function for binary classification. In all layers (except the output), ReLU activation was applied, and the network was trained with the Adam optimizer, with a learning rate of 0.0001.

Table 2 summarizes the configuration parameters used to construct the self-supervised CNN. In Step 2, the emotion recognition model uses the same configuration parameters and layers as the self-supervised CNN, with the exception of the heads, which contain dense neural layers that are fully connected and SoftMax activation.

The configuration of the neural network used to recognize emotions is summarized in Table 3.

### 5.4. Datasets

Three public datasets (AMIGOS, DREAMER, and SWELL) were selected and combined to evaluate the self-supervised learning approach. In general, the datasets contain sensor data and are labeled with affective states. However, each one has distinct characteristics, such as the equipment used to collect the signals, the collection protocol, the stimuli to the participants (e.g., sound or audiovisual), and the emotion model (e.g., discrete or dimensional), among other features. Table 4 presents a summary of the selected datasets with emphasis on the number of classes per category of the available label.

#### 5.4.1. AMIGOS

The AMIGOS dataset [14] was collected to study each individual’s personality, mood, and affective responses based on neurological and physiological signals by exposing 40 participants to multimedia content in two different contexts, alone and in a group of 4 people.

For the execution of this study, participants watched short and long video clips to stimulate emotions. The short video clips had a duration of 250 s, while the long video clips had a duration of 14 min. ECG signals were captured using Shimmer sensors at a sampling frequency of 256 Hz. Three electrodes were installed on the body of each participant, one on each arm and the third one on the inner part of the left ankle. A total of 16 short video clips were shown to each participant, and 4 long video clips were shown to 37 participants, 17 alone and 20 in 5 groups of 4 people.

Regarding emotion labeling, internal labeling was performed, in which participants self-rated their own affective states in arousal (1 to 9) and valence (1 to 9) scores at the end of each video clip.

#### 5.4.2. DREAMER

The DREAMER database [15] consists of EEG and ECG signal data from 23 participants collected during emotion arousal sessions. In the sessions, participants received audio and visual stimuli in the form of film excerpts to produce 9 different affective responses, amusement, excitement, joy, calm, anger, disgust, fear, sadness, and surprise. A total of 18 film clips were shown to each participant, each clip lasting 60 s. In addition, neutral video clips were shown before each film segment to help participants return to a neutral affective state.

Regarding emotion labeling, after each film excerpt, participants responded with their self-assessments in arousal (1 to 5) and valence (1 to 5) scores.

For the execution of this study, ECG signals were collected using a SHIMMER ECG sensor, at a sampling rate of 256 Hz. Three electrodes were installed on the body of each participant, one on each arm and the third one on the inner part of the left ankle.

#### 5.4.3. SWELL

The SWELL database [16] was compiled to study stress and user modeling. ECG signals were collected from 25 participants while performing typical activities such as writing reports, giving presentations, reading emails, and searching for information on the Internet. At the same time, the work environment was altered to include stressful elements such as interruptions by emails and demands regarding the length of time to complete the activity.

For the execution of this study, three affective states were considered: neutral (activity without interruption and without time constraints for completing the activity), time-based stress (30 min for completing the activity), and interruption-based stress (sending a variety of emails, some important and others irrelevant). In relation to emotion labeling, participants reported their self-assessments in arousal (1 to 9) and valence (1 to 9) scores.

The ECG signal was collected using the Mobi TMSI device at a sampling rate of 2048 Hz with electrodes positioned around the heart. One was placed below the right collarbone and the other below the chest, with the ground electrode below the left collarbone.

#### 5.4.4. Data Pre-Processing

To minimize the effects of variations and discrepancies present in the datasets three pre-processing steps were performed on the data. First, the ECG signals present in the SWELL database were re-sampled at 256 Hz so that the sampling rate was like those adopted in the AMIGOS and DREAMER datasets. Next, a high-pass IIR (finite impulse response) filter with a cutoff frequency of 0.8 Hz was used to eliminate low-frequency signals, which are usually produced by electrode polarization. Finally, in the last step, the user-specific z-score normalization of the signals was applied. As a result, the new z-score distributions of the signals from each sensor were centered to have a mean of zero and a standard deviation of one.

After pre-processing, the ECG signals were segmented into a fixed-size window of 10 s without overlapping data, thus avoiding any potential data leakage.

To implement, train, and evaluate the deep neural network architecture, the TensorFlow 2 framework was used and run on a computer with an Nvidia Geforce 1080Ti video card. In addition, the Adam optimizer with a learning rate of 0.001 and 128 batch size was used. In the pre-training stage, 150 training epochs were run, while for the specialization stage, 250 training epochs were run.

As in related work [5], a 10-fold cross-validation was applied to evaluate the model performance for the three datasets. Metrics, such as the accuracy and F1-score, were used. Furthermore, for comparison purposes, results from the same neural architecture trained in a fully supervised manner are presented.

## 6. Results

Table 5 presents the accuracy and F1-score results obtained in the self-supervised pre-training stage of the implemented neural network. These results were obtained considering the combination of training data from the three selected bases. The mean and standard deviation values were obtained from 10-fold cross-validation. The results show that the self-supervised training achieved average values of 99.88% and 97.84% for the accuracy and F1-score, respectively, for all the pretext tasks. The lowest F1-scores were 94.27% and 95.03%, respectively, for the original and scaled pretext tasks.

**Figure 6:**
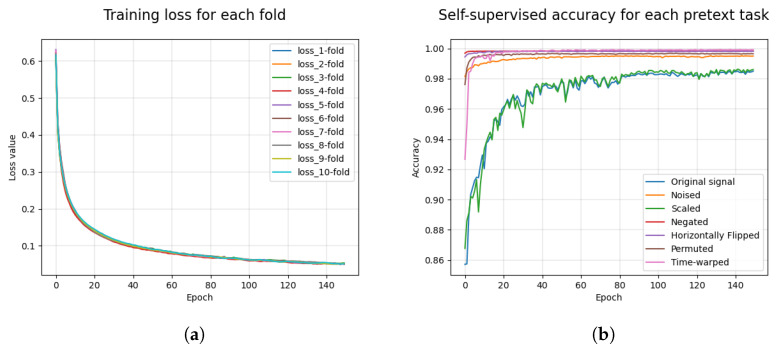
(**a**) Results of the loss functions obtained during the self-supervised training of the implemented neural network. (**b**) Accuracy results obtained in the test set for each of the seven pretext tasks.

Figure 6 shows the obtained values of the model loss function over the self-supervised training for each fold (Figure 6a) and the obtained values of the F1-score on the test set for each pretext task (Figure 6b). These results demonstrate that the model minimized the error on the training set for the 10 folds over the 150 trained epochs. With respect to the accuracy, the noised tasks and original signal did not converge quickly to their highest values, unlike the other tasks.

Table 6 presents the results obtained individually for each of the datasets in the model specialization step for emotion classifications. In addition, the results of the fully supervised training for the same neural network model implemented are presented in order to compare performances.

The results demonstrate that the proposed self-supervised model is effective in emotion classification for three emotion datasets evaluated when compared to the same neural model trained in a fully supervised manner. For AMIGOS, the self-supervised model obtained an accuracy of 80.71% and 77.20% for arousal and valence, respectively. Regarding the F1-score, compared to the supervised model, the self-supervised model showed a positive variance of 13.57% and 16.19% for arousal and valence, respectively. For the DREAMER database, the accuracy results were 69.44% and 66.62% for arousal and valence, respectively. However, the self-supervised model showed a positive F1-score performance over the fully-supervised model of 29.04% and 33.87%, respectively, for arousal and valence. Finally, for the SWELL database, both models achieved the highest accuracy values, with a mean greater than 93%, 93%, and 901% for arousal, valence, and affective state, respectively, and an F1-score greater than 93%, 93%, and 91% for arousal, valence, and affective state, respectively.

### Comparisons with Other Approaches

The tables below show the results of various state-of-the-art methods for emotion recognition tasks reported on the AMIGOS, DREAMER, and SWELL datasets. These results are not directly comparable with one another, nor are they directly comparable with the proposed model. This is because all works used different experiment protocols, such as different segment sizes, different pre-processing steps, different data separations, independent and subject-dependent evaluations, etc. Nevertheless, to give a relative summary of the performances achieved and to compare the proposed model as fairly as possible with the other approaches, the self-supervised model was fully retrained and evaluated for binary emotion recognition (high/low levels of arousal and valence). Therefore, the labels used in the AMIGOS, DREAMER, and SWELL datasets were changed using the mean value of the arousal scale rating and the mean value of the valence scale rating as threshold values to determine a low or high level. The affective state labels used in the SWELL dataset were changed to the no-stress state (“neutral” sessions) and stress state (“time pressure” and “interruptions” sessions).

Table 7 presents the mean accuracies and mean F1 scores for the emotion classifiers evaluated on the AMIGOS dataset. The proposed model achieved an F1-score of 85.29% for arousal and 80.24% for valence. As a result, compared to supervised hybrid deep learning [86], the model outperformed with a positive difference of 5.29% and 4.24%, respectively. Compared to the SSL model by Sakar et al. [5], the model presented lower F1-score values, with negative differences of 2.29% and 2.76% for arousal and valence, respectively. Although both works used the same training approach, hyperparameter optimization and fine-tuning were required to achieve the best classification performance. It should be remembered that the goal of this study was not to determine the most effective classification model, but to highlight the effectiveness of using SSL for emotion recognition.

Table 8 presents the mean accuracies and mean F1-scores for the emotion classifiers evaluated on the DREAMER dataset. The proposed model achieved an F1-score of 70.86% for arousal and 68.49% for valence. Compared with the other studies, the proposed model was significantly lower than the supervised hybrid deep learning model [86] and the SSL model developed by Sarkar et al. [5]. This discrepancy is directly related to the way the signal data were processed. In the hybrid deep learning study, features were extracted from both the ECG signal and the PPG signal, as well as using a more robust architecture. Moreover, Sakar et al. extracted features through two channels of ECG signals (right-arm lead and left-arm lead). In our study, only one ECG channel (the right-arm lead) was used to extract the self-supervised representation and train the emotion classifier model.

Finally, Table 9 presents the mean accuracies and mean F1-scores for the emotion classifiers evaluated on the SWELL dataset. The proposed model outperformed the supervised-transformer-based study [85] for affective state classification and showed comparable results to the SSL model by Sarkar et al. [5] for arousal, valence, and affective state.

## 7. Discussion

In summary, the analysis above showed that the self-supervised approach can achieve results that are on par with or better than fully supervised learning. The findings showed that unlabeled data from three datasets merged to train a model to perform signal transformation classification are able to produce a good and generalizable feature extractor. With the transfer learning, this feature extractor can be reused to train specific models for different target tasks, as shown in the case study for arousal, valence, and affective state.

We emphasize that self-learning can transfer knowledge, which is an important benefit for training networks in real-world settings where there is little or no supervision to learn a model of sufficient quality from scratch. However, a disadvantage would be an increase in computational cost, because the pre-training step requires more time and computational resources to generate the self-supervised training pseudo-labels. Future work to address this issue has recently been investigated through the use of new training methods (e.g., self-adaptive training) [95].

## 8. Conclusions

The problem of recognizing emotions has proven to be a challenging task given the complexity found in the theoretical information on the subject, as well as the various existing approaches and state-of-the-art techniques raised in this research. In this work, a methodology for self-supervised training of deep neural networks for the problem at hand was presented, as well as the advantages of applying this new approach to improve classification rates and reuse the learned representation for new contexts (e.g., database, sensors, representation models).

The self-supervised learning approach enables a representation (feature extractor) to be created from large amounts of unlabeled data and the representation to be reused to specialize models for new problems such as emotion recognition. The experimental results showed that the pretext tasks applied in the pre-training of the neural network were able to provide relevant information in order to obtain a high-level representation. Moreover, the effectiveness of this learned representation was evaluated for the emotion recognition problem via transfer learning and then compared with the fully supervised training approach. We believe that the incorporation of new pretext tasks in the pre-training of the representation extractor model is a promising future direction for self-supervised learning and is beneficial for generalization and performance improvement in emotion recognition problems, especially in those cases where there is a scarcity of labeled data.

## Figures and Tables

**Table 1:** Description of pretext tasks selected for the self-supervised model example.

Pretext Task	Description	Parameter
Noised	This transformation adds random noise to the original input signal.	Signal-to-noise ratio 15
Scaled	This transformation applies a change in the magnitudes of the signal samples from the multiplication of a scalar value.	Scale factor 1.1
Negated	This transformation applies a polarity shift of the signal samples from the inversion function.	-
Horizontally flipped	This transformation applies a change in the temporal order of the samples from an inversion function in time.	-
Permuted	This transformation randomly perturbs samples within a time series by slicing and swapping different segments of the time series to generate a new one.	Permutation pieces 20
Time-warped	This transformation locally extends or deforms the time series by gently distorting time intervals between values.	Stretch factor 1.05, time warping pieces 20

**Table 2:** Specification of the multi-task deep convolutional neural network that was implemented for self-supervised pre-training.

Layer	Specification		Shape
Input	-		2560 × 1
Shared layers	Conv block 1	2 × (Conv1D, 1 × 32, 32, ReLU)	2560 × 32
		Maxpool, 1 × 8, Stride 2	1277 × 32
	Conv block 2	2 × (Conv1D, 1 × 16, 64, ReLU)	1277 × 64
		Maxpool, 1 × 8, Stride 2	635 × 64
	Conv block 3	2 × (Conv1D, 1 × 8, 128, ReLU)	635 × 128
		Global max pooling	1 × 128
Task-specific layers		2 × (Dense, 128 units)	128
		× 7 parallel tasks	
Output		Sigmoid	2
		× 7 parallel outputs	

**Table 3:** Convolutional neural network layer structure and parameters for fully supervised training, as well as transfer learning settings/fine-tuning.

Layer	Specification			Shape
Input	-			2560 × 1
Shared layers	Conv Block 1			
	Conv Block 2			
	Conv Block 3			
Emotion recognition	2 × (Dense, 512 units)			512
Dense layers				
Emotion recognition output	AMIGOS [14]		Arousal	9
			Valence	9
	DREAMER [15]	Softmax	Arousal	5
			Valence	5
	SWELL [16]		Arousal	9
			Valence	9
			Affective state	3

**Table 4:** Summary of the datasets with their respective characteristics such as the number of classes and attributes.

Dataset	Class Group	No. of Classes
AMIGOS [14]	Arousal	9
	Valence	9
DREAMER [15]	Arousal	5
	Valence	5
SWELL [16]	Arousal	9
	Valence	9
	Affective state	3

**Table 5:** Accuracy and F1-score results for the pretext tasks selected for self-supervised training.

Pretext Task	Accuracy	F1-Score
Original signal	98.38%±0.13	94.27%±0.51
Noised	99.44%±0.03	98.05%±0.11
Scaled	98.59%±0.09	95.03%±0.30
Negated	99.88%±0.04	99.59%±0.15
Horizontally flipped	99.83%±0.02	99.40%±0.08
Permuted	99.69%±0.04	98.94%±0.16
Time-warped	99.88%±0.03	99.58%±0.12
**Mean**	99.88%±0.03	97.84%±0.21

**Table 6:** Accuracy and F1-score results for emotion classification of the self-supervised model compared to fully supervised training of the same implemented neural architecture.

Dataset	Group Class	Fully-Supervised		Self-Supervised	
		Accuracy	F1-Score	Accuracy	F1-Score
AMIGOS [14]	Arousal	56.94%±17.30	65.05%±6.65	80.71%±1.79	78.62%±1.97
	Valence	54.44%±15.41	57.98%±9.73	77.20%±1.06	74.17%±1.06
DREAMER [15]	Arousal	42.51%±3.63	38.60%±3.39	69.44%±2.85	67.64%±4.55
	Valence	32.80%±2.06	32.04%±2.63	66.62%±2.97	65.91%±2.96
SWELL [16]	Arousal	92.15%±1.42	92.38%±1.81	93.09%±0.99	93.17%±1.28
	Valence	92.67%±2.31	93.33%±2.21	93.28%±1.09	93.80%±1.11
	Affective State	89.89%±0.89	89.59%±1.05	91.09%±0.79	90.84%±0.81

**Table 7:** Classification results reported by various state-of-the-art works on the AMIGOS dataset.

Study	Approach	Arousal		Valence	
		Accuracy	F1-Score	Accuracy	F1-Score
Hybrid Deep Learning [86]	Supervised	81.89%	80.00%	82.74%	76.00%
Transformer [91]	SSL	88.00%	87.00%	83.00%	83.00%
Proposed Model	SSL	86.00%	85.29%	80.49%	80.24%

**Table 8:** Classification results reported by various state-of-the-art works on the DREAMER dataset.

Study	Approach	Arousal		Valence	
		Accuracy	F1-Score	Accuracy	F1-Score
Hybrid Deep Learning [86]	Supervised	80.68%	77.00%	80.43%	78.00%
CNN [5]	SSL	85.90%	85.90%	85.00%	84.5%
Proposed Model	SSL	71.27%	70.86%	70.24%	68.49%

**Table 9:** Classification results reported by various state-of-the-art works on the SWELL dataset.

Study	Approach	Arousal		Valence		Affective State	
		Accuracy	F1-Score	Accuracy	F1-Score	Accuracy	F1 Score
Transformer [85] ^1^	Supervised	−	−	−	−	71.60%	74.20%
CNN [5]	SSL	96.70%	95.40%	97.30%	96.9%	93.30%%	92.40%
Proposed Model	SSL	96.94%	96.87%	95.58%	95.58%	95.10%	94.84%

^1^ Arousal and Valence results were not reported by the authors.

## Data Availability

AMIGOS [14], DREAMER [15], and SWELL [16] datasets are used for training and testing the proposed method. These datasets are freely available.
